# A new Otogelin ENU mouse model for autosomal-recessive nonsyndromic moderate hearing impairment

**DOI:** 10.1186/s40064-015-1537-y

**Published:** 2015-11-25

**Authors:** Carole El Hakam Kamareddin, Laetitia Magnol, Veronique Blanquet

**Affiliations:** Univ. Limoges, INRA, UMR 1061, Unité de Génétique Moléculaire Animale, Faculté des Sciences et Techniques, 123, Avenue Albert Thomas, 87060 Limoges, France

**Keywords:** Animal model, *N*-ethyl-*N*-nitrosourea mutagenesis, Otogelin, Vestibular balance defect, Deafness, Hearing impairement

## Abstract

Approximately 10 % of the population worldwide suffers from hearing loss (HL) and about 60 % of persons with early onset HL have hereditary hearing loss due to genetic mutations. Highly efficient mutagenesis in mice with the chemical mutagen, ethylnitrosourea (ENU), associated with relevant phenotypic tools represents a powerful approach in producing mouse models for hearing impairment. A benefit of this strategy is to generate alleles to form a series revealing the full spectrum of gene function in vivo. It can also mimic the range of human mutations and polymorphisms for HL. In the course of a genome ENU mutagenesis program, we selected a new mouse model for hearing defect based on a dysmorphological screen. We identified by gene mapping the mutation responsible for this phenotype and characterized it at the histological level of the inner ear and evaluated the vestibule by following the recommendations of the standard operating procedures, IMPReSS. We have identified and characterized a new recessive allele of the otogelin gene, *Otog*^*vbd/vbd*^, due to a homozygous one base pair substitution at the splice donor site of intron 29. This mutation leads to a frame-shift and a premature stop codon. We observed a decrease in the amount of sensory cells in the maculae of *Otog*^*vbd/vbd*^ mice as well as an apparent drastically decreased density to almost absence of the otoconial membrane. Compared to *Otog*^*tm1Prs*^ and twister, the two other existing otogelin alleles, the detailed analysis of *Otog*^*vbd/vbd*^ revealed that these mice share some common behavioural characteristics either with *Otog*^*tm1Prs*^ or twister whereas the fine vestibular phenotype and the hearing defect are different. Our results emphasize the importance of detecting and characterizing a new allele of a gene in order to get comprehensive information about the gene function.

## Background

Hearing loss is one of the most common public health issues and comes in the third place for the major physical condition after the arthritis and heart disease. About 2–3 of every 1000 children have hearing difficulties or deafness (http://www.hearingloss.org). Two types of hearing impairment (HI) are commonly known based on the defective part of the hearing organ. They are traditionally classified as conductive or sensorineural HI. The conductive HI refers to defects in the outer or middle ear. Such a loss yields a mild to moderate HI and it is usually treated with either medical or surgical intervention. It also indicates a normal inner ear activity. In the other hand, sensorineural HI refers to a problem in the inner ear or along the auditory pathway; it’s also known as nerve-related hearing loss. In contrast, such loss of hearing yields a mild to profound HI and could not be completely solved. There is also the mixed HI; it refers to a conductive and a sensorineural loss occurring at the same time. While the conductive component may be treated, the sensorineural one remains permanent.

Hearing loss (HL) can be due to genetic or environmental causes or a combination of both. The majority of human hereditary HL (HHL) cases are isolated or associated with only a vestibular dysfunction and so classified as non-syndromic HL (NSHL); but HHL may be also accompanied with other abnormalities (syndromic hearing loss; SHL). More than 50 % of NSHL cases are inherited in an autosomal recessive manner. So far, more than 80 loci have been described, and for 60 of these, the mutated genes have been identified (Van Camp and Smith 2015 in http://hereditaryhearingloss.org).

Model organisms such as the mouse whose genes, regulatory regions, and genome structure are remarkably similar to those of humans provide powerful tools to unravel the functional and evolutionary complexities of the human genome (Brown and Hardisty 2003; Oliver et al. 2007; Nguyen and Xu 2008), and so for the understanding of the molecular mechanisms of hearing. In the most of HHL cases, a single mutation in a single gene is responsible for HL. In addition, different mutations in the same gene may lead to both syndromic and non-syndromic HHL as well as different onset times which may vary from birth to old age (http://hereditaryhearingloss.org), these occurring human mutations could be more closely modelled by mutants identified through ENU-based screens (Nolan et al. 2002; Aigner et al. 2007). ENU (*N*-ethyl *N*-nitrosourea) is one of the most effective alkylating mutagens in mice and it is known to randomly induce single-point mutations at a rate of ~ 100-fold higher than the rate of spontaneous mutations (Justice et al. 1999; Balling 2001; Concepcion et al. 2004; Cordes 2005; Gondo 2009). Such chemical mutagenesis induces different pedigrees of mice, each one is potentially harbouring a different DNA mutation for one of many genes contained in a chromosome region which are subjected to a broad range of tests to identify the mutant phenotype (Hrabé de Angelis et al. 2000; Soewarto et al. 2003; Davisson et al. 2012). A wide genome screening based on polymorphic markers followed by DNA sequencing allows the identification of the causal mutation responsible for the detectable phenotype. Furthermore, ENU mutagenesis is particularly a valuable methodology to recover an allelic series of point mutations for any gene enabling a more acute analysis of gene function (Graw et al. 2004; Quwailid et al. 2004; Augustin et al. 2005; Sachs et al. 2007; Gondo 2009, 2010). Thus ENU with the other transgenic methods are able to create an allelic series of different types of mutations.

In this report, we describe the characterization of a new ENU mouse model for autosomal-recessive nonsyndromic hearing impairment presenting a mutation in the *Otog* gene (DFNB18B, MIM 614945). Otogelin is one of the non-collagenous N-glycosylated proteins specific to the acellular membranes covering the six sensory epithelial patches of the inner ear (Cohen-Salmon et al. 1997). In the vestibule, Otog is required for the anchoring of the otoconial membrane and the cupula to the neuroepithelia. In the cochlea, Otog appears to be involved in organizing the fibrillar network of the tectorial membrane and it likely has a role in determining the resistance of this membrane to sound stimulation (Simmler et al. 2000a; El-Amraoui et al. 2001). We called the new allele *vbd* for vestibular balance defect. This mouse model displays a behavioural and hearing phenotype. This third allele after twister (twt) and *Otog*^*tm1Prs*^ previously characterised in literature, showed partly characteristics of the two previous one. Our results highlight the importance of mutations allelic series in the functional analysis of a specific gene.

## Results

### Both vestibular and auditory functions are impaired in Otog^vbd/vbd^ mice

Mutant offspring are clearly identifiable at 4 days of age by their uncontrolled postural response. When placed on their backs (righting reflex), *Otog*^*vbd/vbd*^ mutants stayed supine without trying to rotate to an upright position unlike wild-type mice which recovered their posture within 10 s. After 2 weeks of age, more than 90 % of *Otog*^*vbd/vbd*^ mice had a one side tilted head and showed an impaired balance. Balance problems were more obvious when animals were held by the tail and dropped onto a soft surface. Whereas wild-type animals always land on their feet, mutants fell on their back or on their sides. Such behaviour indicated a saccular defect (Sondag et al. 1998). Mutants showed also abnormal response for the elevated platform test. Moreover, about 10 % of *Otog*^*vbd/vbd*^ mutants showed a circling behaviour since the postnatal day 10 (P10) that persists throughout adulthood. When tested in the swimming test, none of the mutant mice are able to swim or float properly, but they remain submerged, completely disoriented, while turning under water. They were rapidly removed from water to prevent drowning. This severe vestibular phenotype was fully penetrant. On contrary, wild-type mice came immediately to the water surface and maintained a horizontal bodyline at the surface. *Otog*^*vbd/*+^ animals responded normally to all the tests.

**Fig. 1:**
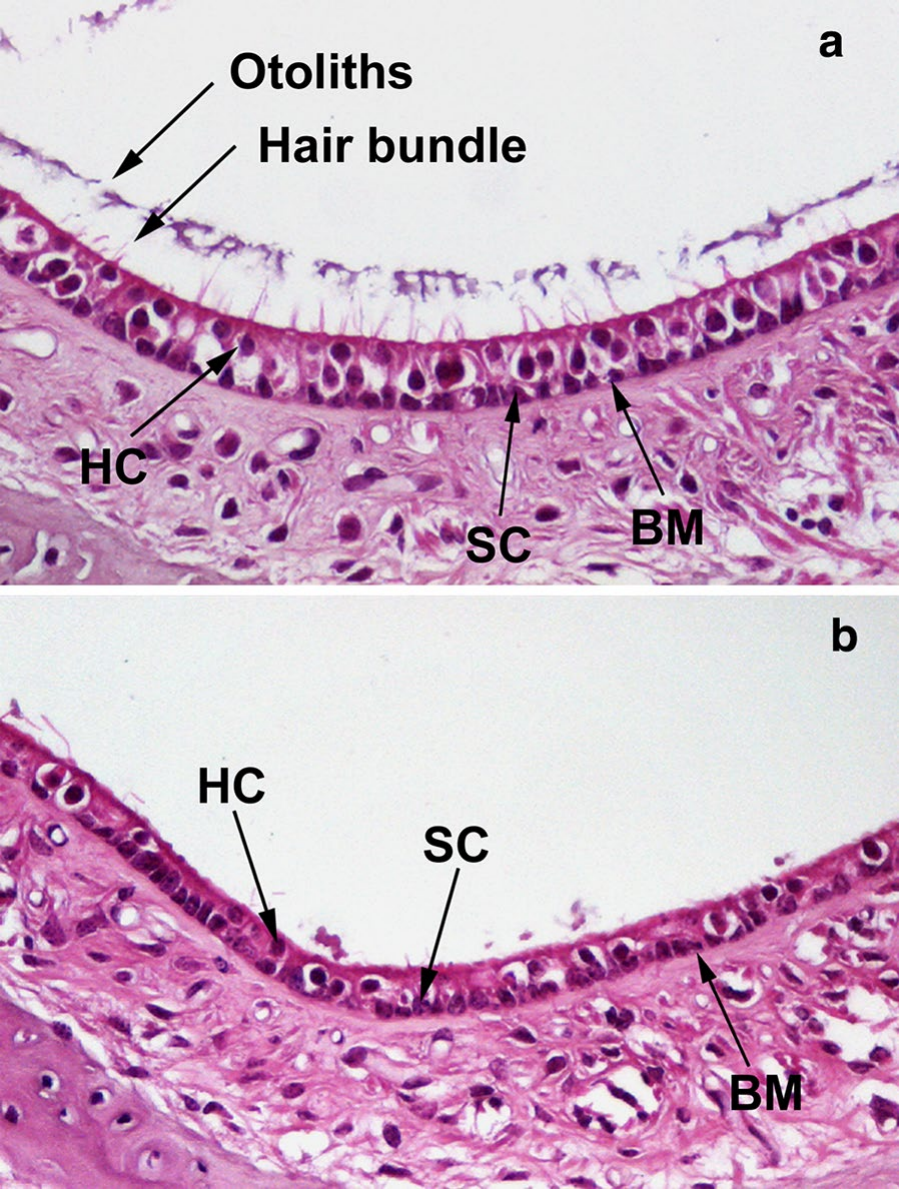
Comparative sections of the maculae in wildtype and *Otog* ^*vbd/vbd*^ adult mice (P90). On saccular maculae of the control mouse (**a**), the otoconial membrane (layer of otoconia + hair bundles) is visible. In contrast, in *Otog* ^*vbd/vbd*^ mice (**b**), only few remnants of the otoconial membrane (probably remnants of hair bundles) are present. The supporting cells (SC) at the base of the maculae are preserved. It seems that sensory cells (HC) are affected, as shown by the drastic decrease in the number of hair bundle. *BM* basement membrane. (Magnification: ×40)

**Fig. 2:**
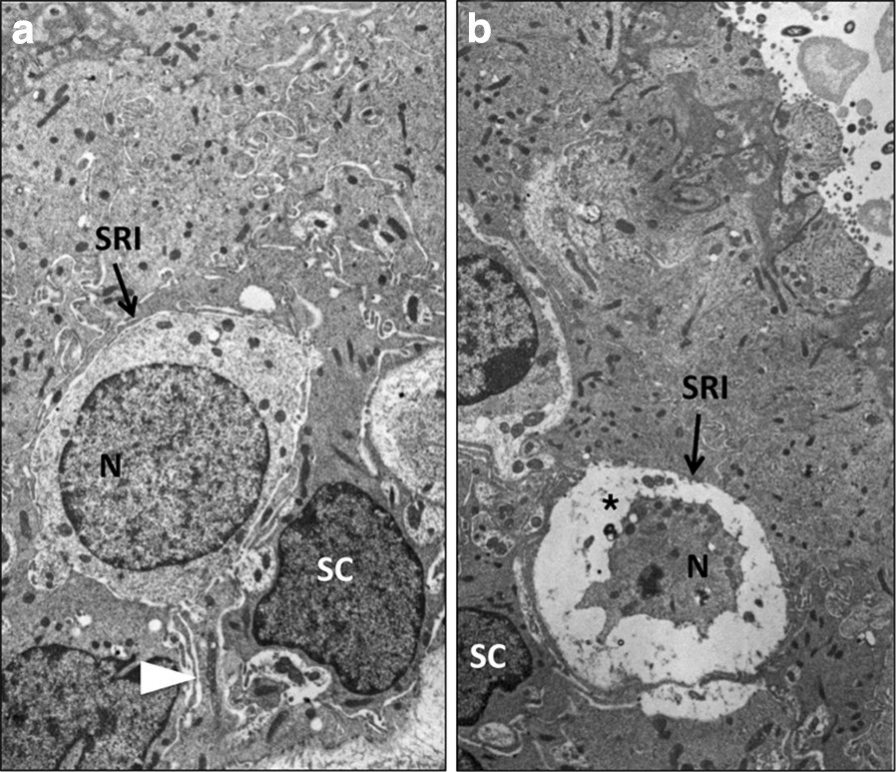
Electromicroscopy analysis of type I sensory cells (SRI). **a** Wildtype SRI showing the afferent nerve calyx (*arrow head*) embedded in supporting cells (SC). In *Otog* ^*vbd/vbd*^ (**b**), some type I cell are shrunken (*asterisk*). N: nucleus

In order to a further characterization of the developmental defect induced in *Otog*^*vbd/vbd*^ mice, histological analysis of the inner ear morphology was performed on P4 mutants and wild-type mice. Constantly, anomalies in the macula were revealed in mutants. This latter presented a drastically decreased density to almost an absence of the otoconial membrane, which recovers the macula (Fig. 1). Only very few remnants of the membrane were visible. The stereocilia of the sensory hair cells were much less numerous, consistent with a decreased number of sensory hair cells (Fig. 1). Electronmicroscopy (EM) allowed us to confirm these data. In mutants, it was also noticeable that numerous type I hair cells of the maculae have a shrunken nucleus (Fig. 2). The biominerals themselves, otoconia, were preserved as well as the supporting cells in the expected density. In the crista ampullaris, the cupula was normally present as in the control and mutants, except in older *Otog*^*vbd/vbd*^ mice (>P100) where the cupula were detached. The cochlea histological analysis of *Otog*^*vbd/vbd*^ mice did not reveal any gross morphological anomaly. We observed the three normal compartments of the cochlea as well as the organ of Corti (data not shown).

We verified the auditory function of *Otog*^*vbd/vbd*^ mice compared to their wildtype littermates at various ages, ranging from 3–18 weeks by the Preyer reflex. At the weaning age, out of 20 *Otog*^*vbd/vbd*^ mutants, two animals did not react to the test, exhibiting an abnormal Preyer reflex, while a normal response was observed in the 18 remaining tested mice. All the mice were tested again between 8 and 12 weeks of age, 10 more mutants became progressively unresponsive. Finally, at 16 weeks of age, none of the mutant mice reacted anymore. All controls or heterozygous mice always exhibited a normal Preyer reflex.

### Genetic mapping and sequencing

**Fig. 3:**
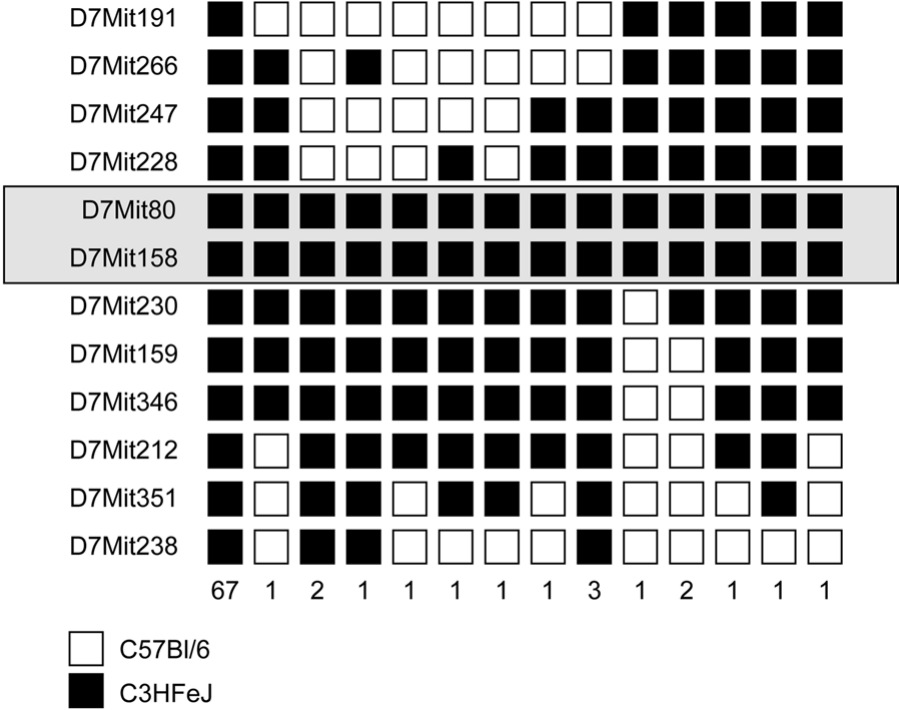
Genetic mapping of *vbd* mutation. Haplotype analysis of 84 mutant mice derived from an outcross-intercross strategy. *Markers* are shown on chromosome 7. The *black* and *white boxes* represent the C3HeB/FeJ and the C57BL/6J allele, respectively. The number of mice with the same allele distribution is noted at the *bottom* of each *column*. The *grayed box* indicates the chromosomal interval bearing the *vbd* mutation

**Fig. 4:**
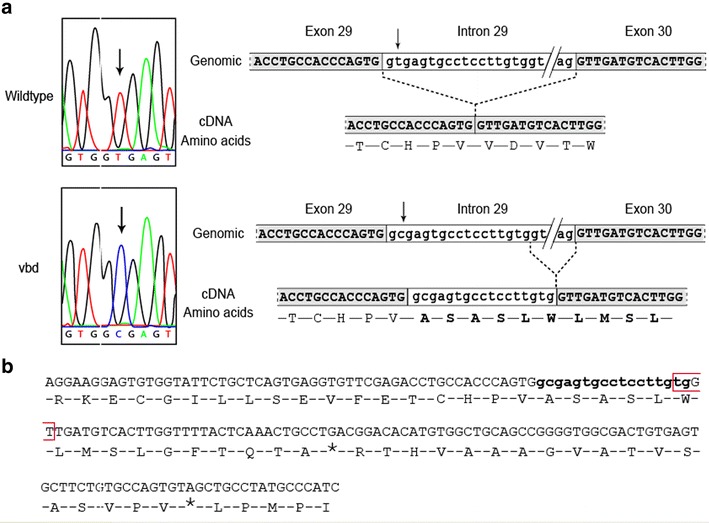
Sequence analysis spanning the *vbd* mutation site. **a** (*Left*) Sequence chromatograph of the *otog* gene at the level of the splicing site exon 29/intron 29 showing the T > C transition (*arrows*); (*right*) partial genomic sequence of the region spanning exon 29 to exon 30 with the corresponding cDNA and amino acid sequence. *Top*: wildtype; *bottom*: *Otog* ^*vbd/vbd*^ mutant. **b** cDNA sequence surrounding the *vbd* mutation showing the insertion of 17 bp (*lowercase* bases in *bold*) including a new splicing site (*open red box*). The resulting reading frame shift leads to a premature stop codon in the amino acid sequence between VWFD 3 and VWFD 4 domain (uniprot)

The *vbd* mutation was mapped by PCR based microsatellite analysis of 85 *Otog*^*vbd/vbd*^ mutant mice produced by an outcross-intercross breeding strategy. Linkage was obtained with markers on mouse chromosome 7 in an interval of 16.85 cM, between the markers D7Mit266 (16.67 cM) and D7Mit159 (33.52 cM). There was no evidence of any linkage elsewhere in the genome. To further narrow the region, additional markers were used allowing us to define a critical region of 5.83 cM between markers D7Mit228 (25.61 cM) and D7Mit230 (31.44 cM) (Fig. 3). Based upon these mapping results and the known genes in this genomic region, we selected the *otogelin* gene as a good candidate gene for the *vbd* mutation. Sequence analysis of *Otog* in *Otog*^*vbd/vbd*^ mutant mice revealed no change in the coding region but a T36351 > C36351 transition at the splice donor site of intron 29 (numbering from the first base of the translation codon at the genomic level) (Fig. 4a). By RT-PCR and sequencing, we showed that this splice site mutation leaded to an activation of a downstream cryptic splice-site within intron 29, causing an insertion of 17 bases of intron 29 sequences into the mature RNA (Fig. 4b). This leaded to a frame-shift of translation inducing an addition of 16 new amino acids at the carboxyl-terminus, before an encountered in-frame stop translation codon, and truncating the otogelin protein (Fig. 4c). Genotyping all mice by sequencing has verified the mutation. This mutation was not detected in various stocks of inbred strains including C3HeB/FeJ, C57BL/6J, 129S2, BALB/cJ and DBA/2J (data not shown). The *vbd* phenotype resulted from a recessive mutation in the *otogelin* gene giving rise to a new chemically induced allele that we named *Otog*^*vbd/vbd*^.

## Discussion

**Fig. 5:**
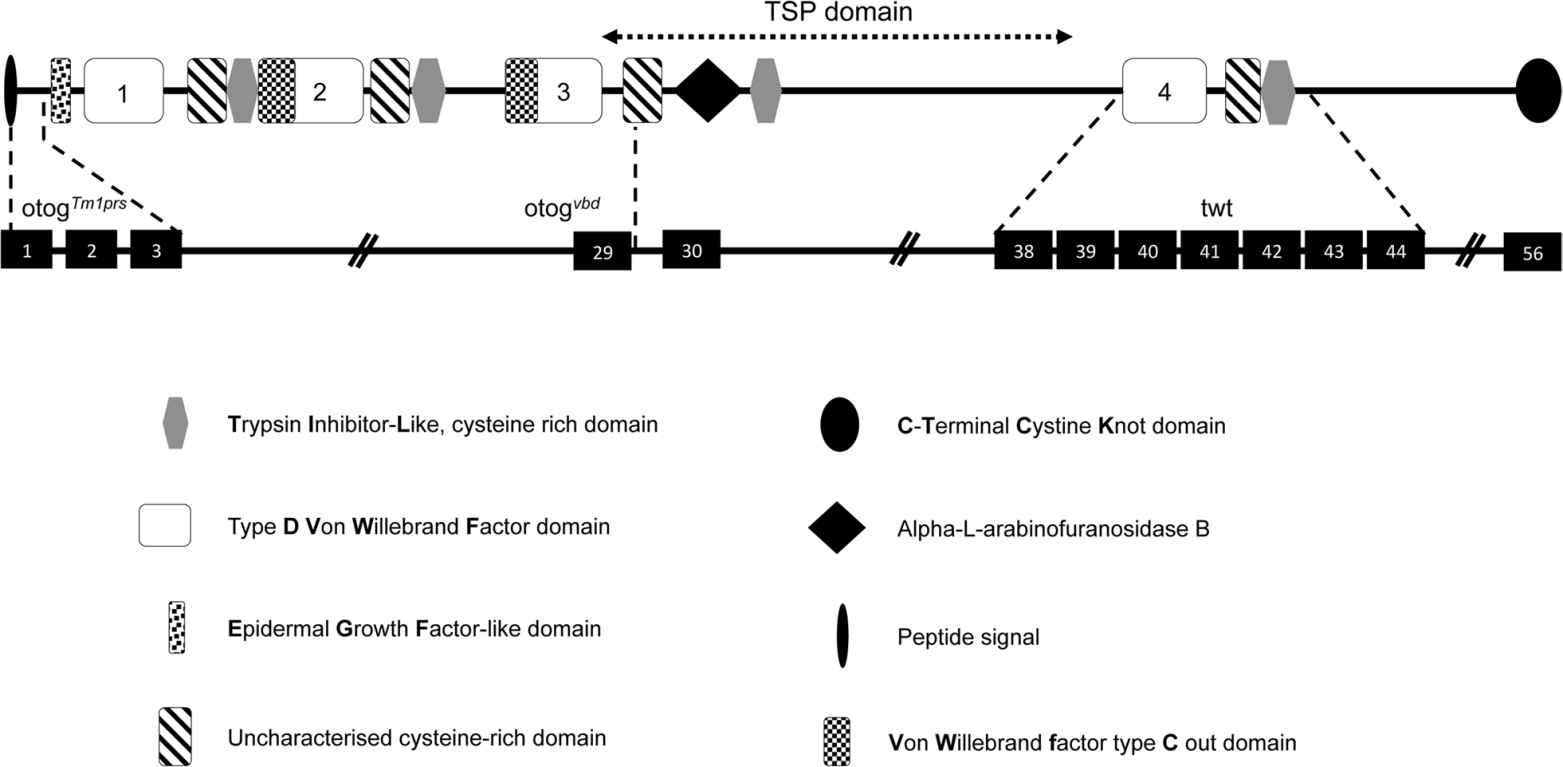
Schematic representation of OTOG protein. The emplacement of the different domains of OTOG protein are shown (*top*). The positions of the three mutations and the corresponding exons where the mutations occur are shown at the *bottom*. otog^*vbd*^ (T > C transition at the level of the splicing site exon 29/intron 29 of the *otog* gene), otog^*tm1prs*^ (replacement of the majority of exon 1 and all of exons 2 and 3 by the lacZ gene fused in frame with the translation initiation codon of *Otog)*, and twister (spontaneous recessive mutation showing a discrete rearrangement within the 3′ part of the *Otog* locus, between exons 38 and 44 not precisely identified)

In this present study, we have identified and characterised a new recessive ENU-induced mutation in the otogelin gene, named *Otog*^*vbd/vbd*^, inducing a moderate hearing impairment. *Otog*^*vbd/vbd*^ could be added to the two other existing otogelin alleles twister (twt) and. *Otog*^*tm1Prs*^. Twt mice (B6/C3H background) have a spontaneous recessive mutation in *Otog* gene showing a discrete rearrangement within the 3′ part of the *Otog* locus, between exons 38 and 44 not precisely identified leading to the absence of *Otog* transcript whereas *Otog*^*tm1Prs*^ mice (129S2 background) present a null mutation with a complete gene inactivation due to the replacement of the majority of exon 1 and all of exons 2 and 3 by the lacZ gene fused in frame with the translation initiation codon of *Otog* using gene targeting (Lane 1981; Simmler et al. 2000a, b). The exact location of the three mutations is shown on Fig. 5.

Whilst some full loss of function mutations do have such a GT to GC change, it is worth noting that 0.5–1 % of introns have GC as normal splice donor. Using RT-PCR followed by electrophoresis on WT and vbd total RNA from inner ear to amplify the region between exon 28 and exon 30, we did not observe any normal splicing in vbd mice (data not shown). Protein expression was not carried out because the only antibodies available and working on Western blot are directed against the Threonine, Serine and Proline rich region TSP domain (position 1451–2036a.a) of the otogelin protein. Unfortunately, this area is absent in our *Otog*^*vbd/vbd*^ mutant as well as in the *Otog*^*tm1Prs*^ mice, making impossible to test the presence of a functional truncated protein in these mutants. El-Amraoui et al. (2001) have described the expression of otogelin in the cochlea and the vestibular apparatus, in all the neuroepithelial supporting cells. These acellular structures play a crucial role in the auditory and balance mechanotransduction processes.

The detailed analyses of *Otog*^*vbd/vbd*^ mice reveal that they share some common behavioural characteristics either with *Otog*^*tm1Prs*^ or twister mice at the same age. Firstly, the circling behaviour observed in twister mice is also present in *Otog*^*vbd/vbd*^ mutants since P10 whereas it is not observed in *Otog*^*tm1Prs*^ mice. Secondly, *Otog*^*vbd/vbd*^ mutant mice as well as *Otog*^*tm1Prs*^ animals are unable to swim properly and remain submerged unlike twister mice which do not sink. However, the three of them adopt an underwater circling behaviour. Finally, the new allele described here shows a typical waltzing behaviour, i.e. a circling and an abnormal swimming behaviour. This observation suggests a more pronounced behavioural phenotype in *Otog*^*vbd/vbd*^ than in *Otog*^*tm1Prs*^ and twt. For the vestibular phenotype, the three mutants show abnormal otolithic membrane morphology at P4: in twt and *Otog*^*tm1Prs*^, the otoconial membrane is present but partially or fully detached from the sensory epithelium in the saccule and the utricle (Simmler et al. 2000a, b); however, *Otog*^*vbd/vbd*^ mutant show a drastically decreased density to almost absence of the otoconial membrane in the maculae. The analysis of the three mutants shows the importance of the C-terminal part of the protein in the anchoring of the otoconial membranes and the cupula to the underlying neuroepithelia in the vestibule. By their inability to swim, the *Otog*^*vbd/vbd*^ and *Otog*^*tm1Prs*^ mice seem to have a more severe behavioural phenotype than the twister mutant. As histological analyses do not reveal any morphological differences between *Otog*^*vbd/vbd*^ and twt, in addition, as shown in Fig. 5, the region encompassing exons 30–37 is present in twt but absent in the other two mutants, thus the loss of this region could play a role in the underwater behaviour.

The identification of novel mutant alleles is important for understanding critical functional domains of a protein and establishing genotype—phenotype correlations. Additional alleles, apart from the null alleles obtained by homologous recombination are invaluable in revealing previously unknown aspects of the gene function. Otogelin-like (Otogl) is similar to otogelin in structure (40 % of amino acid sequence identity in conserved regions) and localization. It is found in the acellular membranes of the inner ear, and the mutations of this gene result in moderate hearing loss with no difference in phenotype based on *Otog* mutations (Cohen-Salmon et al. 1999; Yariz et al. 2012; Bonnet et al. 2013).

So far, there is an increasing number of multiple mutant alleles in a wide variety of genes due to spontaneous or chemically induced mutations allowing not only the discovery of deafness genes mutation but also the molecular mechanisms of hearing and the pathogenesis of deafness as for the protein tyrosine phosphatase receptor type Q gene (PTPRQ; MIM 603317) (Goodyear et al. 2003) and lipoxygenase homology domains 1 gene (LOXHD1; MIM 613072) (Grillet et al. 2009), and cadherin-23 gene (CDH23; MIM 605516) (Schwander et al. 2007; Manji et al. 2011). Another example is for the transmembrane cochlear-expressed gene 1 (TMC1; MIM 606706). A dominant or a recessive mutation of the human ortholog, TMC1 causes a progressive hearing loss (DFNA36) or a profound congenital deafness (DFNB7/B11), for which Beethoven (Bth) and deafness (dn) are mouse models, respectively (Kurima et al. 2002; Vreugde et al. 2002).These examples and more others highlight the importance of mutant mouse in this process (Rhodes et al. 2004; Longo-Guess et al. 2007; Lewis et al. 2009; Carpinelli et al. 2013; Miller et al. 2013), providing valuable insight into gene functions. Identifying an allelic series is an efficient tool to generate clinically relevant mouse models (Vernersson Lindahl et al. 2013). The vertebrate inner ear is an organ of extraordinary functional characteristics and structural intricacy. Mutant mice have been instrumental in elucidating the function and mechanisms of the inner ear (Friedman et al. 2007; Hardisty-Hughes et al. 2010).

## Conclusion

*Vbd* is a new recessive ENU mutation of *Otog* gene characterized by a typical waltzing behaviour and a hearing impairment sharing some common behavioural characteristics either with *Otog*^*tm1Prs*^ or twister, the other two allele of *Otog*. Our results emphasize the importance of detecting and characterizing a new allele of a gene in order to get comprehensive information about the gene function. After the international efforts to generate conditional alleles for every protein coding gene in the mouse genome by high throughput conditional gene targeting and trapping (the International Knockout Mouse Consortium (IKMC); http://www.mousephenotype.org/about-ikmc, the ongoing International Mouse Phenotyping Consortium (IMPC; http://www.mousephenotype.org) will undoubtedly discover and ascribe biological function to new genes/alleles involved in hearing impairment.

## Methods

### Mice

The ENU induced-*Otog*^*vbd*^ line was generated and maintained on pure C3HeB/FeJ (C3Fe) genetic background as previously described in the Munich genome wide ENU recessive mutagenesis program (Hrabé de Angelis et al. 2000; Soewarto et al. 2003). The *vbd* mutation was also outcrossed with C57BL/6J (B6) mice then maintained by intercrosses. B6C3Fe F2 animals were used for the genetic mapping, the phenotypic and histological analyses. All mice were bred and housed under controlled conditions (21 °C, 12-h light/12-h dark cycle) with free access to standard mouse chow (RM1 (P) 801151, Special diets services) and tap water. All animal experiments were performed according to European directives (86/609/CEE and 2010/63/UE) and approved by the Committee on Ethics of Animal Experiments from the Author’s Institution, “Comité Régional d’Ethique de l’Expérimentation Animale” of the Limousin region (n° 10-2014-10). According to the European Directive 210-63-EU, mice were observed daily for the general health status and mortality scoring. We carried out all tests on mice between 3 and 18 weeks of age, using 5 animals of each genotype: Wildtype (*Otog*^+*/*+^), heterozygous (*Otog*^*vbd/*+^) and homozygous (*Otog*^*vbd/vbd*^).

### Vestibular evaluations

All protocols follow the recommendations of the standard operating procedures EMPReSS, actually IMPReSS (http://www.mousephenotype.org/impress). Mice were subjected to a battery of vestibular evaluations, including observation of their circling behavior and head-tilting, the righting reflex, the elevated platform test, the Preyer reflex and the swim ability test. The Preyer reflex, a startle reflex of the pinna, was evaluated by using a supra-threshold sound burst delivered from a calibrated clickbox (20 kHz, 96 dB SPL, MRC Institute of Hearing of Research). The reflex was considered positive when a rapid movement of the whole body of the animal was clearly noticed. For the swimming ability test, mice were placed individually in a large mouse cage filled with at least 15 cm depth of water (25 °C) for a maximum of 60 s, and were scored for the time spent with their nose and tail maintained above the water surface.

### Histological analyses

Histological characterization of *Otog*^*vbd/vbd*^ mice was conducted by Frimorpho, Inc. (Fribourg, Switzerland). Briefly, after anaesthesia, each mouse was perfused with Karnovky-fixative. The inner ears were isolated, decalcified in EDTA, and then dehydrated through graded alcohol series. For a global analysis, organs were embedded in paraffin and subjected to classical procedures for hematoxylin-eosin staining. Pictures were taken with a Leica digital camera. For electron microscopy, the inner ears were embedded in epon 812. Semithin sections were cut with a Reichert Ultramicrotome mounted on glass slides and stained with Toluidine Blue. Ultrathin-sectioning was performed on the same instrument, followed by lead citrate contrasting and uranyl acetate staining. Transmission electron-microscopy was made on a Philips EM-10. Photographic plates were from Ilford.

### Genetic and comparative gene sequence analysis

A genome-wide linkage analysis was performed on 84 B6C3Fe *Otog*^*vbd/vbd*^ mice using a panel of 60 MIT polymorphic markers between C3HeB/FeJ and C57Bl/6 as described previously (Besson et al. 2005). Centimorgan positions for markers were determined based on MGI database (http://www.informatics.jax.org). For sequencing, specific primer pairs were designed in the flanking region of each *Otog* gene exon. The amplicons were generated from the DNA of two *Otog*^*vbd/vbd*^ mutants as well as two controls (parental C3HeB/FeJ strain). They were sequenced using ABI Big Dye terminator chemistry and analysed on an ABI 310 automated sequencer (Applied Biosystems, Foster City, CA, USA). After the identification of the mutation, all animals were then genotyped by sequencing.

### Isolation of total RNA, RT-PCR and sequencing of the transcripts

Total RNA was extracted from the inner ear of 2 weeks old mice using Trizol reagent (Life Technologies) and Qiagen RNAeasy columns. RT-PCR was carried out using two primers set: otog1-fw: (5′_GTGGGCCTCTGTGGGAACTT_3′); otog1-rev: (5′_GGGGTGTCCGCCAGTCAATG_3′) otog2-fw: (5′_ATGCGGACCCCAGAGAACCT_3′); otog2-rev: (5′_TGTCCGTCAGGCAGTTTGAG_3′). cDNA products were sequenced using ABI Big Dye terminator chemistry.
